# The Doctor's Indifference

**Published:** 1916-06-24

**Authors:** 


### THE DOCTOR'S INDIFFERENCE.

ONE of our lay contemporaries has recently published an article on "The Higher Indifference," meaning by this term "not an effort to avoid thinking, but an effort made by thinking to avoid pain." The distinction is emphasised in order to exclude the species of mental protection from painful emotion which consists in a refusal to contemplate subjects apt to give rise to such emotion. This "lower" variety of indifference is, of course, practised more or less by everyone, and has as its typical example the person who refuses to read the paper which contains bad news. Here is the deliberate effort to escape pain by the avoidance of thinking. The "higher" form, our author tells us, is often attained by doctors. Face to face with ills, some of which he cannot cure or even assuage, his sympathy with the patient is perfectly sincere, but it does not interfere with his sleep or his appetite, his enjoyment of life or his scheme of the universe. He is neither careless nor callous, but he is not less happy nor more hard-hearted than his fellows. And this position the doctor attains—such is the suggestion—by will power.

The medical profession will certainly not quarrel with the sketch thus presented. It is appreciative and sympathetic, and it is dictated by understanding and good will. We quite agree that the doctor must, unless he would wear himself out, be protected in some way or other from the emotional assaults to which his work exposes him. But we are not quite sure that our contemporary is right when its contributor finds this protection in a deliberate action of the will. Our own inclination would be to say rather that it is a result—even a necessary result—of knowledge, and that it is strictly parallel with less technical experiences. One lesson taught by medical work is the inevitable progress of certain processes which are not less natural because we choose to call them disease; another is that while there are methods by which in some degree these processes may be retarded such methods are limited both in number and value. While much is uncertain in medicine, the two propositions just stated are illustrated daily. Similarly any educated man with a fair acquaintance with natural science knows well that Nature is "red in tooth and claw," and he knows exactly how much or how little he can do to redeem the situation. In the one instance and in the other the observer is face to face with facts, and from such an experience is born the "higher indifference," not as a resolution of the will, but from the consciousness either that the situation is beyond relief or that everything possible is being done to redress it. It is this sure conviction which makes calm possible, the certainty that it is useless to kick against the pricks. With a wavering balance there may be anxiety and even distress, but a decree fixed leaves the mind steady. When Dr. Johnson was asked what he would do were one of his friends apprehended for an offence for which the penalty was hanging, he replied, "I should do what I could to bail him and give him any other assistance; but if he were once fairly hanged I should not suffer." When fate has spoken the final word—and the doctor knows more of this than anyone else—the knowledge in itself is a protection against mental anguish.

